# Determinants of Pre- and Post-Procedural Neurological Assessment, and Outcome of Carotid Endarterectomy or Stenting

**DOI:** 10.3390/jcm13144177

**Published:** 2024-07-17

**Authors:** Michael Kallmayer, Christoph Knappich, Felix Kirchhoff, Bianca Bohmann, Vanessa Lohe, Shamsun Naher, Hans-Henning Eckstein, Andreas Kuehnl

**Affiliations:** Department for Vascular and Endovascular Surgery, Klinikum rechts der Isar, Technical University of Munich, 81675 Munich, Germany; michael.kallmayer@mri.tum.de (M.K.);

**Keywords:** carotid artery stenosis, carotid endarterectomy, carotid artery stenting, neurological assessment, quality assurance, national registry

## Abstract

**Background:** The German–Austrian guideline on the treatment of carotid stenosis recommends specialist neurological assessment (NA) before and after carotid endarterectomy (CEA) or carotid artery stenting (CAS). This study analyzes the determinants of NA and the association of NA with the perioperative rate of stroke or death. **Materials and Methods:** This study is a pre-planned sub-study of the ISAR-IQ project, which analyzes data from the nationwide German statutory quality assurance carotid database. Patients were classified as asymptomatic (group A), elective symptomatic (group B), and others (group C: emergency (C1), simultaneous operation (C2), and other indications (C3)). The primary outcome event (POE) of this study was any in-hospital stroke or death. Adjusted odds ratios for pre- and post-NA and the POE were calculated using multivariable regression analyses. **Results:** We analyzed 228,133 patients (54% asymptomatic, 68% male, mean age 72 years) undergoing CEA or CAS between 2012 and 2018. Age and sex were not associated with the likelihood of pre-NA or post-NA. The multivariable regression analysis showed an inverse association between pre-NA and POE (adjusted odds ratio (aOR) 0.47; 95% CI 0.44–0.51, *p* < 0.001), and a direct association of post-NA and POE (aOR 4.39; 95% CI 4.04–4.78, *p* < 0.001). **Conclusions:** Pre- and postinterventional specialist NA is strongly associated with the risk of any in-hospital stroke or death after CEA or CAS in Germany. A relevant confounding by indication or reversed causation cannot be ruled out. Nevertheless, to improve the quality assurance of treatment, the NA recommended in the guideline should be carried out consistently.

## 1. Introduction

Arteriosclerotic stenosis of the extracranial part of the carotid artery is the cause of around 15% of all ischemic strokes. The therapeutic approaches available to prevent these strokes are optimal drug therapy alone or in combination with revascularization by means of carotid endarterectomy (CEA) or stent-assisted carotid angioplasty (carotid artery stenting, CAS). While hemodynamically effective (in-stent) restenosis rarely leads to complications requiring revision, the preventive effects are mainly countered by arterio–arterial embolic complications such as transient ischemic attack (TIA) or post-procedural strokes [1,2]. Based on previously published data from the German carotid quality assurance registry [3,4], the recommendations on maximum tolerable rates of stroke and death after CEA or CAS have been reduced from ≤3% to ≤2% in asymptomatic patients, and from ≤6% to ≤4% in symptomatic patients [5]. To ensure the quality of treatment and improve patient care, international guidelines, and in particular the European and German–Austrian guideline, recommend specialist neurological assessment (NA) both before and after CEA or CAS [2,5,6]. “All patients with a carotid stenosis should undergo clinical neurological examination” (expert consensus) “and the peri-interventional stroke and death rate should be examined by a specialized neurologist” (strong recommendation, level-of-evidence 2a) [3,5].

Beyond that, the European guideline emphasizes the importance of a multidisciplinary team approach including involvement of neurologists, stroke physicians, vascular surgeons, interventional cardiologists, or radiologists [2]. Independent neurological assessment before and after carotid interventions is recommended to audit peri-procedural risks ([2], recommendation 7). However, the underlying evidence to support this resource-depleting approach is old and sparse [7,8,9]. Nevertheless, an independent neurological assessment of all CEA and CAS patients seems reasonable to avoid misclassification or (intended) self-reporting bias of outcome events, which may impact the validity of quality assurance, especially in unmonitored registries. To date, outcomes (perioperative stroke and death) recorded in German statutory quality assurance for the treatment of carotid stenosis have been based on self-reporting by the physicians or the department that treated the patient being monitored, with or without independent NA before (pre-NA) or after the procedure (post-NA).

Therefore, the aim of this study was to analyze determinants of NA and to assess the association between pre-/post-procedural NA and the perioperative rates of stroke and death following CEA or CAS.

## 2. Methods

The present analysis is a pre-planned sub-study of the ISAR-IQ project (Integration and Spatial Analysis of Regional, site-specific, and patient-level factors for Improving Quality of treatment for carotid artery stenosis).

### 2.1. Data Source

The study is based on the nationwide German statutory quality assurance database operated by the Institute for Quality Assurance and Transparency in Healthcare (Institut für Qualitätssicherung und Transparenz im Gesundheitswesen: IQTIG, according to § 136 SGB V). Because of legal obligations, the data collection covers all CEA operations and CAS procedures. The legal basis for scientific use of quality assurance data is § 137a paragraph 10 SGB V. The study was approved by the ethics committee of the Medical Faculty, Technical University of Munich (Reference Number 107/20S). The analysis was conducted according to Good Practice of Secondary Data Analysis guidelines. As this is an observational study using routinely collected health data, RECORD reporting guidelines were applied [10]. All data are saved on IQTIG servers, according to the respective data protection regulations. Data access was only permitted using controlled remote data processing (CRDP). Essential methods regarding CRDP have been established in other studies and recently published [11,12,13,14]. The study protocol was submitted to the IQTIG and the G-BA (German Federal Joint Committee: Gemeinsamer Bundesausschuss) during the application procedure but was not published separately. Further details of legal basis have already been published [15]. Data on carotid revascularization procedures in Bavarian hospitals are statutorily collected by the Bayerische Arbeitsgemeinschaft Qualitätssicherung (BAQ) and passed to the IQTIG. For data protection reasons, the unique hospital identifier is pseudonymized when the data are transmitted to the IQTIG, so that linking the patient-level data with those of levels 2 (hospital data) and level 3 (regional data) was only possible for Bavaria.

### 2.2. Case Selection and Grouping

**Figure 1:**
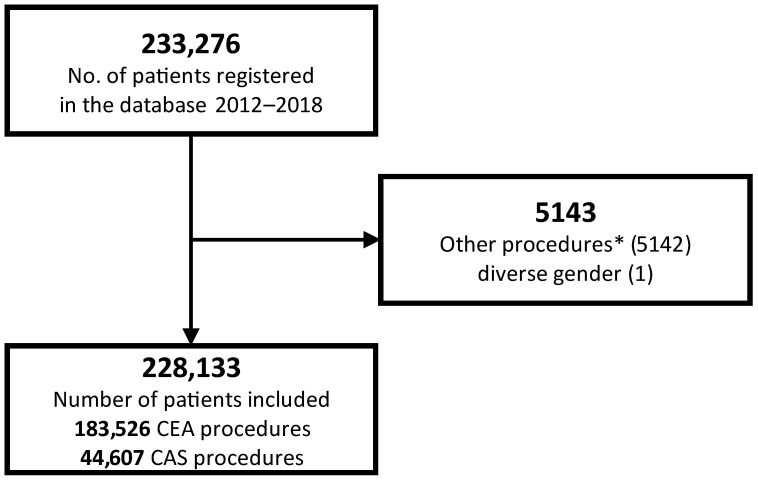
Patient flowchart. * = Excluding combined/converted procedures (CAS and CEA), and CAS procedures performed for the primary purpose to gain access for an intracranial intervention.

All patients receiving either CEA or CAS for carotid stenosis between 2012 and 2018 were included. Patients with CAS procedures for the primary purpose of gaining access for an intracranial intervention or combined/converted CEA/CAS procedures were excluded. See Figure 1 for flowchart. The cases were grouped as follows. Group A: asymptomatic patients without carotid-related cerebral or ocular symptoms within the last 6 months. Group B: symptomatic patients receiving elective treatment. Group C: symptomatic patients receiving emergency treatment for, e.g., crescendo transient ischemic attack (TIA) or stroke-in-evolution (C1); simultaneous cardiac, aortic, or peripheral vascular operations (C2); or other procedures (C3). Patients were classified according to NA before the procedure (pre-NA) and NA after the procedure (post-NA). This also reflects the main structure of the tables. Both variables are directly coded in the quality assurance database.

### 2.3. Study Outcomes

The primary outcome event (POE) was any stroke or death until discharge from hospital. Secondary outcomes were major stroke or death, any postprocedural stroke, or all-cause death, each until hospital discharge. Stroke symptoms were graded as minor (modified Rankin Scale, mRS 0–2) or major (mRS 3-5). No information on cause of death is provided in the registry.

### 2.4. Statistical Analyses

Categorical variables are presented as absolute numbers and percentages. If not stated otherwise, continuous variables are presented as median with first (Q1) and third (Q3) quartiles. Odds of pre- or postprocedural NA, and odds of POE were modeled using a logistic regression model. To account for confounding, age, sex, American Society of Anesthesiologists (ASA) stage, presence of symptoms, and ipsilateral and contralateral degree of stenosis were entered as fixed-effect factors. To model risk of POE, pre- and post-procedural assessment by a specialist in neurology was additionally included as a fixed factor. Model specification and variable selection were done a priori according to a pre-specified analysis plan based on literature research and expert knowledge.

R version 3.2.1 (R Foundation for Statistical Computing, Vienna, Austria) was used for data processing and statistical analysis, with extension packages to calculate cross-classified tables and multivariable regression analyses. Variable codes were extracted from the codebooks provided by the IQTIG and harmonized over the time period from 2005 to 2018. Graphic processing of the data was performed using R. For all tests, a two-tailed level of significance of α = 5% was used.

## 3. Results

### 3.1. Baseline Characteristics of Patients

In total, 228,133 cases were included. The median age was 72 years, and two-thirds of patients were male (68%). Of all patients, 73% received pre-NA, and 63% post-NA (Table 1). Most patients were classified as ASA III (64%) followed by ASA stage I/II (33%). Ipsilateral degree of stenosis was 70–99% in 91% of cases, and 2% had an occlusion of the internal carotid artery. Two-thirds of patients had no or only mild contralateral stenosis (68%), and 6% suffered contralateral occlusion. In most cases, patients were asymptomatic (59%). About one-quarter (26%) presented with amaurosis fugax, transient ischemic attack (TIA), or stroke. In 15% of cases, patients suffered from crescendo-TIA or stroke-in-evolution, or were treated due to other symptoms or under special conditions such as combined cardiac surgery. See Table 1 for a differentiated presentation with regard to pre-NA and post-NA status.

### 3.2. Perioperative Management and Treatment

Most of the symptomatic patients treated electively (not for emergency reasons) received CEA or CAS between 3 and 7 days after the qualifying event (38%). Eleven percent were treated within 2 days, and 50% after 8 days or later. See Table 2 for further details. The median center annual caseload for all procedures was 37 (Q1-Q3, 12-73), for CEA 22 (2-56) and for CAS 2 (0-14), each based on all participating centers. Of all patients, about two-thirds (67%) were admitted Monday through Wednesday. Only 3% were admitted on Saturdays, but the rate of pre-NA was highest on that day (91%). Pre-procedurally, patients received duplex ultrasound in 69%, transcranial duplex ultrasound in 21%, computed tomography angiography in 38%, and magnetic resonance angiography in 34%.

### 3.3. Neurological Assessment by Indication for Treatment

Overall, 80% of patients received CEA, whereas 20% were treated with CAS (Table 3). The highest proportion of endovascular treatment was found in group C2 (simultaneous procedures, 45% CAS), and in emergencies (group C1, crescendo-TIA and stroke-in-evolution, 39% CAS). Patients treated with CAS underwent more frequent preoperative and postoperative specialist neurological examinations than patients treated with CEA, regardless of the indication for treatment. Asymptomatic CAS patients were assessed in 71% before (CEA: 58%) and in 62% after the procedure (CEA: 53%). See Table 3 for further details regarding NA by indication for treatment.

### 3.4. Hospital and Regional Characteristics (Bavarian Data Only)

About two-thirds of patients were treated in maximum care hospitals (37%), and main care hospitals (29%). Pre-NA was basically performed in about 70% of cases, with only primary care providers and other hospitals having a lower rate at about 50% (see Supplemental Table S1 for further details). Most (78%) of the cases were performed in municipally owned hospitals, 17% in privately owned, and 6% in non-profit hospitals. Median hospital size was more than 500 beds. With regard to pre-NA and post-NA, there were no relevant differences in the number of beds between the departments of vascular surgery and neurology. Half of the patients (51%) were treated in hospitals with emergency rooms available on-site. A stroke unit certified by the German Stroke Society was available on-site in 32%. Only 17% of patients were treated in vascular centers, which are certified by the German Vascular Society. Most patients received CEA or CAS in hospitals in large cities (47%), but 46% of care was also provided in rural counties or sparsely populated counties. Regarding the German Index of Socioeconomic Deprivation (GISD, standardized), the index ranged from 0.32–0.36 (Supplemental Table S1).

### 3.5. Neurological Assessment by Outcome

Overall, there were 6254 POE, which results in an average risk of stroke or death of 2.74% (Table 4). POE risk was significantly higher when pre-NA was performed (RR 1.48, 95% CI 1.39–1.57, *p* < 0.001), but even higher when post-NA was performed (RR 3.46, 95% CI 3.23–3.71, *p* < 0.001). In general, CAS patients who experienced the primary outcome were more likely to be assessed by a neurologist than CEA patients. The same was basically true for patients receiving pre-NA, except in group C2 (simultaneous procedures).

Asymptomatic patients who received post-NA were significantly more likely to suffer a POE compared to patients who received no post-NA (CEA: 2.19% vs. 0.61%, *p* < 0.001; CAS: 2.37% vs. 0.72%, *p* < 0.001). The same was true for electively treated symptomatic patients (group B) in whom the POE risk was significantly higher when post-NA was performed (each *p* < 0.001; Table 4). See Supplemental Table S2 for details on secondary outcomes.

### 3.6. Multivariable Regression Analysis of Determinants of Pre-NA and Post-NA

**Figure 2:**
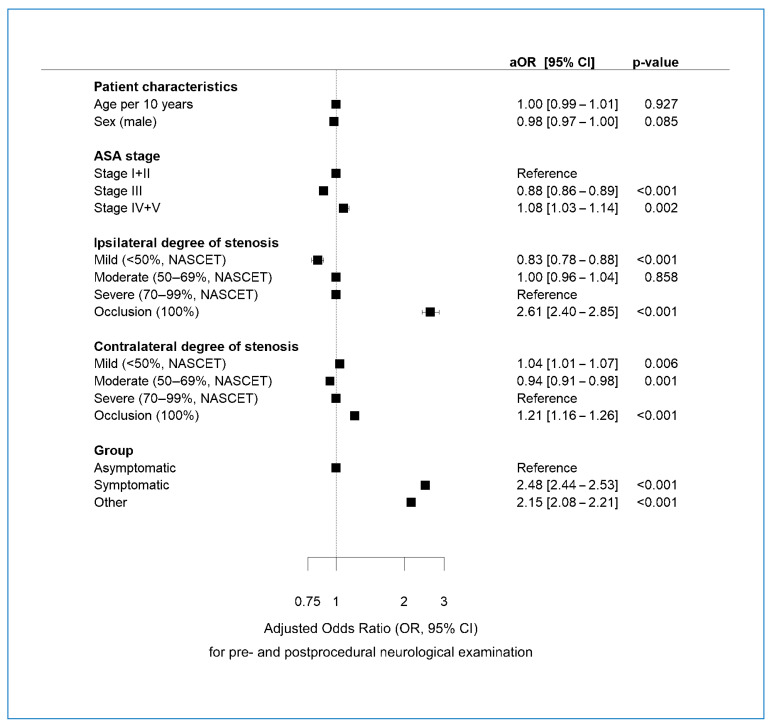
Multivariable regression analysis of determinants associated with pre- and postprocedural neurological examination. aOR = adjusted odds ratio. ASA: American Society of Anesthesiologists. NASCET = degree of stenosis by the North American Symptomatic Carotid Endarterectomy Trial.

Multivariable regression results revealed that the following determinants were significantly associated with a higher likelihood of receiving guideline-compliant pre-NA and post-NA (Figure 2): ASA stage IV/V vs. stage I/II, ipsi- or contralateral carotid occlusion, mild contralateral stenosis, and symptomatic status. In contrast, ASA Stage III, mild ipsilateral stenosis, and moderate contralateral stenosis were associated with lower-than-average likelihood of receiving both pre- and post-NA. Age and sex were not associated with the likelihood of receiving pre- or post-NA. See Supplemental Figures S1 and S2 for the multivariable analysis differentiated by pre-NA and post-NA.

### 3.7. Multivariate Regression Analysis of Factors Associated with POE

**Figure 3:**
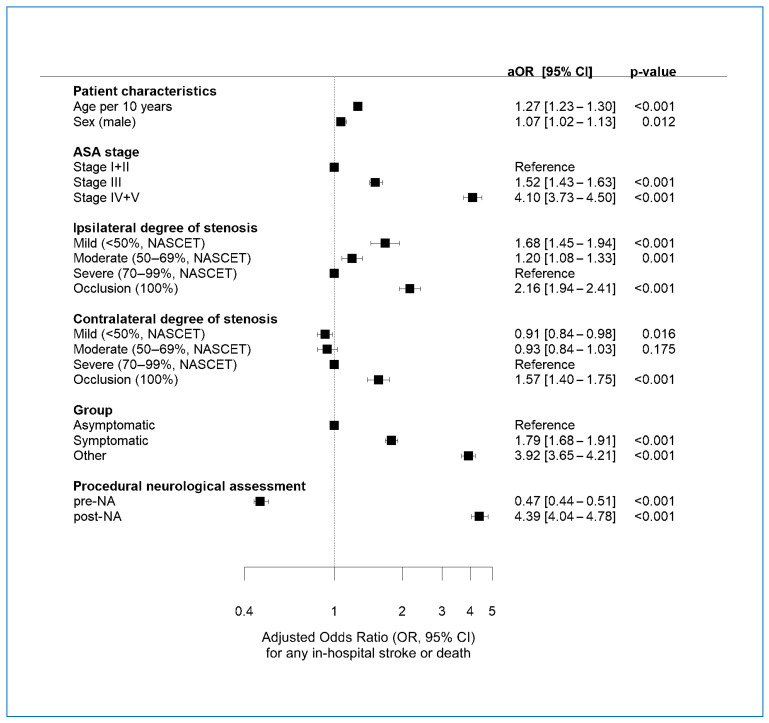
Multivariable regression analysis of factors associated with any stroke or death. aOR = adjusted odds ratio. ASA: American Society of Anesthesiologists. NASCET = degree of stenosis by the North American Symptomatic Carotid Endarterectomy Trial.

The following factors were significantly associated with higher POE risk (Figure 3): higher age, male sex, higher ASA stage, ipsilateral occlusion or stenosis <70%, contralateral occlusion, and symptomatic status or other symptoms. Contralateral stenosis <50% was significantly associated with lower POE risk. Performing pre-NA was significantly associated with lower POE risk, whereas post-NA was significantly associated with higher risk of POE. The latter two associations were the strongest among all factors analyzed.

## 4. Discussion

To our knowledge, this is the first nationwide evaluation of independent NA before and after CEA and CAS. This study shows that pre- and postoperative specialist NA is strongly associated with the risk of a perioperative stroke or death after CEA and CAS in Germany. While pre-NA is associated with lower risk, post-NA is related to higher POE risk. A relevant confounding-by-indication or reverse causation cannot be ruled out and must be considered when interpreting the results of quality assurance analyses. Nevertheless, to avoid information bias in quality assurance, this study provides further real-world evidence for guideline authors, and reinforces existing recommendations that pre- and post-NA should be carried out consistently before and after CEA or CAS. Data collection for this study was prospective and unselected, and as documentation of all CEA and CAS cases is statutory by law in Germany, the data set is virtually complete, which minimizes the risk of selection bias.

Both the European and the German–Austrian guidelines on carotid stenosis give specific recommendations regarding periprocedural NA. Although only on the level of ‘expert consensus’, it is stated that all patients should be evaluated by an experienced neurologist regardless of symptom status before CEA or CAS (strong recommendation) [2,5]. In contrast, this analysis reveals a considerable lack of guideline adherence, with nearly 30% of patients receiving no NA before CEA or CAS. Age and sex of the patients did not affect the implementation of the guideline recommendation, whereas multimorbid patients (ASA stage ≥ 4) and symptomatic patients were assessed more often before CEA or CAS compared to healthier patients and asymptomatic cases. With respect to postinterventional assessment, an astonishing 38% of all CEA and CAS patients did not receive post-NA, contrary to guideline recommendation [2,5]. In patients who received post-NA, the primary endpoint was documented over 3.7 times more frequently than in patients without post-NA. The association between post-NA and POE risk was the strongest association in the multivariate analysis. The detection of even mild new neurological symptoms is probably the result of the precise and thorough assessment by a specialist in neurology. However, the data do not allow the conclusion that patients who were not assessed by a neurologist were in part under-diagnosed. Nevertheless, it should be kept in mind that a neurological evaluation, ideally performed by the same neurologist, is more likely to reveal slight changes in symptom severity (before vs. after) than when performed by a physician who is not specially trained. Conversely, this does not mean that patients who were not evaluated by a neurologist did not have new symptoms. This possible bias is reflected in significant differences in outcome rates for asymptomatic and electively symptomatic patients treated with CEA when NA was performed before surgery.

The same relationship, although not significant, was found for asymptomatic CAS patients. Therefore, we must assume that a significant proportion of symptomatic patients are missed when NA is not performed. This demonstrates the importance of a thorough examination prior to intervention, as the time window and criteria for indication are highly dependent on symptom status.

Regarding structural requirements, the German–Austrian carotid guideline recommends that neurological and vascular medical services experienced in the treatment of cerebral ischemia should be available 24/7 to provide CEA or CAS (expert consensus) [5]. CEA patients were more frequently assessed before and after intervention in centers with lower annual caseloads, whereas CAS patients were more likely to be assessed in centers with higher annual volume. It should also be mentioned that clinics without a neurologist (small units in which only the surgeon performs postprocedural neurological outcome after CEA or CAS) may deliver better results in the quality assurance (“observer bias” [16]) than large university hospitals where the outcome is examined more frequently, and independently (“diagnostic access bias” [17]). Therefore, the guideline recommendation for routine neurological examination (pre-NA and post-NA) seems to be very reasonable.

Interestingly, the American Society for Vascular Surgery guideline does not mention a recommendation to the topic of NA at all [1]. The underlying available evidence for the recommendations in the European and German–Austrian guidelines is unfortunately limited. Randomized controlled trials (Level 1 Evidence [18]) addressing the impact of pre-NA or post-NA during in-patient treatment of carotid stenosis with CEA or CAS do not exist. However, the effect of an independent audit of morbidity and mortality of carotid endarterectomy was evaluated in a systematic review of studies on symptomatic carotid stenosis in 1995 [7]. In that review, 50 studies reporting on 15,959 procedures evaluated the combined risk of stroke and death. The overall risk was 5.6% (4.4–6.1%) and varied from 2.3% (1.8–2.7%) when single surgeons reported their own results, to 7.7% (5.0–10.2%) when postoperative assessment was performed by a physician or neurologist [7]. Similar findings were reported from the Pro-CAS registry. In 2079 CAS procedures, the combined stroke and death risk was 3.5% when a neurologist had assessed the patient before and after the procedure, compared to 1.3% in 1188 patients who had been assessed by the interventionalist [8]. These studies show that one of the main biases in all non-externally supervised registries or observational studies relates to the common practice of self-reporting of outcomes. In order to avoid self-reporting bias, all protocols of randomized controlled trials (RCTs), starting from NASCET [6] to ACST-2 [19] require mandatory independent NA before and after the treatment. A neurologist or, if not available, an independent and board-certified neuroscientist or physician other than the one conducting the study, was also accepted [6]. RCTs are the best study design to control for known and unknown confounders by ensuring that the groups differ only in terms of the assessed intervention. In contrast, in observational studies, as in this evaluation of the German Carotid Registry, it is important to control for confounding factors, because unknown confounders may bias the outcome of any study.

In the multivariable analysis, this study showed a strong inverse association of pre-NA with the POE risk, and a strong direct association between post-NA and the POE risk. Therefore, identifying this strong confounder cannot be over emphasized, as missing it could lead to blurring an existing connection (“negative confounding”) or to faking a non-existent connection (“positive confounding”). Nevertheless, other biases can influence the outcome rates of a registry. The observed higher rates of stroke and death, when post-NA was carried out, could suggest that a post-NA increases the risk of a POE. This assumption would be a confounding-by-indication. In turn, suspicion of new neurological symptoms after the procedure may lead to higher rates of post-NA and thus, more post-NA leads to more detected POE [20]. This so-called “diagnostic suspicion bias” [21] must be considered, as we may see more outcomes if we take a closer look (e. g. specialist neurological and independent assessment). On the other hand, reverse causation could also occur when a POE has definitely and obviously occurred and post-NA is performed consecutively to assess, e.g., the severity of symptoms or to plan further treatment. It seems reasonable that patients with symptoms are more likely to be neurologically assessed than those who show no obvious post-interventional symptoms. In summary, the outcome rates of the quality assurance program may therefore be underestimated. Additionally, the German statutory quality assurance only covers the in-hospital period. Subsequently, this also means that the maximum in-hospital stroke or death rates (the unbiased actual 30-day rates) recommended by the German guideline [5] of 2% in asymptomatic patients and 4% in electively treated symptomatic patients may be an ambitious goal.

### Limitations and Strengths

This is a secondary data analysis and thus, all shortcomings of observational studies using routine data must be considered. These limitations were discussed in detail elsewhere [22,23] and will be summarized here.

First, the study design was retrospective and observational. Because patients were not randomized for the different procedural techniques and adjunct measures, selection bias as well as confounding by indication is possible. This implies that all results need to be interpreted as associations rather than causal relationships.

Second, follow-up data covered only the in-hospital period. Because most of the perioperative events presumably occur within the first days after CAS, detection bias is considered to be low and evenly distributed across the groups.

Third, all data in the database are self-reported by the attending physicians, and reporting bias cannot be ruled out. However, data reports were reviewed by the regional offices for quality assurance (Landesgeschäftsstellen für Qualitätssicherung) and the occurrence of suspect data induced a process of structured dialogue to clarify abnormalities systematically. Nonetheless, underreporting of adverse events is theoretically probable and might be the reason for the overall low rates of perioperative stroke or death reported in this registry (1.5% in asymptomatic patients group A, and 2.9% in electively treated symptomatic patients group B). For comparison, in a systematic review, the 30-day risk of stroke or death was reported to be 3.3% in asymptomatic and 7.6% in symptomatic patients [15,24]. Although underreporting cannot be ruled out, any potential information bias can be considered homogeneous among the variables analyzed in this study.

Fourth, residual confounding cannot be excluded, because some possible confounders were not collected (e.g., co-medication, history of external neurological examination, indication for pre-NA or post-NA). Last, no information on cause of death is provided in the registry.

In contrast, the strength of the study is the legally binding, nationwide, complete data collection of all CEAs and CAS procedures performed in Germany and standardized documentation. It is a comparatively large cohort of real-world data with a low risk of selection bias. In addition, the research question relates to the important methodological issue of quality assurance and how this can be further improved.

## 5. Conclusions

This study showed that NA was carried out inconsistently and too infrequently in Germany. The results underline the importance of pre- and post-interventional NA, which was strongly associated with the risk of in-hospital stroke or death after CEA or CAS in the German quality assurance database. Despite a small selection bias, relevant confounding by indication or reverse causality cannot be excluded and must be taken into account in future statistical risk adjustment within the framework of statutory external quality assurance. To further improve the quality assurance of treatment, the guideline recommendations on peri-interventional NA should be consistently implemented.

## Figures and Tables

**Table 1:** Baseline characteristics of patients by neurological assessment.

	Overall (Column-%)		Pre-Operative Neurological Assessment				Post-Operative Neurological Assessment			
			Yes		No		Yes		No	
**No. of cases (%)**	228,133	(100)	165,459	(72.5)	62,674	(27.5)	14,2619	(62.5)	85,514	(37.5)
**Age (M, Q1/3)**	72	(65–78)	72	(64–78)	72	(65–77)	72	(64–78)	72	(65–77)
**Male sex**	155,532	(68)	113,045	(72.7)	42,487	(27.3)	97,280	(62.5)	58,252	(37.5)
**Side of treatment (right)**	114,427	(50)	82,454	(72.1)	31,973	(27.9)	70,950	(62.0)	43,477	(38.0)
**ASA stage**										
Stage I+II	73,744	(33)	53,269	(72.2)	20,475	(27.8)	46,521	(63.1)	27,223	(36.9)
Stage III	144,104	(64)	104,015	(72.2)	40,089	(27.8)	88,751	(61.6)	55,353	(38.4)
Stage IV+V	8161	(4)	6563	(80.4)	1598	(19.6)	5932	(72.7)	2229	(27.3)
**Ipsilateral degree of stenosis °**										
Mild (<50%)	4076	(2)	2723	(66.8)	1353	(33.2)	2488	(61.0)	1588	(39.0)
Moderate (50–69%)	11,715	(5)	9543	(81.5)	2172	(18.5)	7803	(66.6)	3912	(33.4)
Severe (70–99%)	208,265	(91)	149,515	(71.8)	58,750	(28.2)	128,794	(61.8)	79,471	(38.2)
Occlusion (100%)	4077	(2)	3678	(90.2)	399	(9.8)	3534	(86.7)	543	(13.3)
**Contralateral degree of stenosis °**										
Mild (<50%)	155,231	(68)	112,612	(72.5)	42,619	(27.5)	97,587	(62.9)	57,644	(37.1)
Moderate (50–69%)	31,375	(14)	22,370	(71.3)	9005	(28.7)	18,815	(60.0)	12,560	(40.0)
Severe (70–99%)	27,159	(12)	19,593	(72.1)	7566	(27.9)	16,851	(62.0)	10,308	(38.0)
Occlusion (100%)	14,368	(6)	10,884	(75.8)	3484	(24.2)	9366	(65.2)	5002	(34.8)
**Neurological symptoms ^&^**										
Asymptomatic (A)	122,363	(59)	73,425	(60.0)	48,938	(40.0)	66,580	(54.4)	55,783	(45.6)
Amaurosis fugax (B)	12,375	(6)	9854	(79.6)	2521	(20.4)	7937	(64.1)	4438	(35.9)
Transitory ischemic attack (B)	23,257	(11)	20,422	(87.8)	2835	(12.2)	15,933	(68.5)	7324	(31.5)
Any stroke (B)	18,787	(9)	17,659	(94.0)	1128	(6.0)	13,910	(74.0)	4877	(26.0)
SIE or crescendo-TIA (C1)	9962	(5)	9206	(92.4)	756	(7.6)	8289	(83.2)	1673	(16.8)
Simultaneous operations (C2)	6851	(3)	4390	(64.1)	2461	(35.9)	4063	(59.3)	2788	(40.7)
Other symptoms (B + C3)	13,754	(7)	10,812	(78.6)	2942	(21.4)	9317	(67.7)	4437	(32.3)
**Morphological characteristics**										
Ulcerated plaque	22,630	(10)	17,732	(78.4)	4898	(21.6)	14,654	(64.8)	7976	(35.2)
Aneurysm	1418	(1)	1023	(72.1)	395	(27.9)	927	(65.4)	491	(34.6)
Coiling	1648	(1)	1333	(80.9)	315	(19.1)	1094	(66.4)	554	(33.6)
Multiple lesions	6354	(3)	5213	(82.0)	1141	(18.0)	4638	(73.0)	1716	(27.0)
Others	7770	(3)	5761	(74.1)	2009	(25.9)	5147	(66.2)	2623	(33.8)

Unless otherwise indicated, percentages of total values refer to the row. M, Q1/3 = median with first/third quartile. ASA: American Society of Anesthesiologists. ° = North American Symptomatic Carotid Endarterectomy Trial (NASCET) degree of stenosis. ^&^ = Group A (asymptomatic patients), Group B (symptomatic patients receiving elective treatment), Group C (symptomatic patients receiving emergency treatment (C1), simultaneous operations (C2), other procedures (C3)). SIE = stroke in evolution. TIA = transient ischemic attack.

**Table 2:** Perioperative management and diagnostic measures by neurological assessment.

	Overall (Column-%)		Pre-Operative Neurological Assessment				Post-Operative Neurological Assessment			
			Yes		No		Yes		No	
**Time interval from symptom onset**										
0–2 days	8273	(11)	7453	(90.1)	820	(9.9)	6456	(78.0)	1817	(22.0)
3–7 days	27,597	(38)	25,465	(92.3)	2132	(7.7)	20,555	(74.5)	7042	(25.5)
8–14 days	15,333	(21)	13,909	(90.7)	1424	(9.3)	11,017	(71.9)	4316	(28.1)
15–180 days	22,316	(30)	18,228	(81.7)	4088	(18.3)	13,903	(62.3)	8413	(37.7)
**Day of week (admission)**										
Monday	58,066	(25)	40,450	(69.7)	17,616	(30.3)	35,355	(60.9)	22,711	(39.1)
Tuesday	48,287	(21)	34,656	(71.8)	13,631	(28.2)	30,047	(62.2)	18,240	(37.8)
Wednesday	45,356	(20)	31,797	(70.1)	13,559	(29.9)	27,334	(60.3)	18,022	(39.7)
Thursday	35,653	(16)	25,773	(72.3)	9880	(27.7)	21,872	(61.3)	13,781	(38.7)
Friday	17,575	(8)	14,470	(82.3)	3105	(17.7)	12,228	(69.6)	5347	(30.4)
Saturday	7125	(3)	6512	(91.4)	613	(8.6)	5672	(79.6)	1453	(20.4)
Sunday	16,071	(7)	11,801	(73.4)	4270	(26.6)	10,111	(62.9)	5960	(37.1)
**Preoperative diagnostics**										
Duplex ultrasound	156,306	(69)	110,954	(71.0)	45,352	(29.0)	93,828	(60.0)	62,478	(40.0)
Transcranial Doppler	46,925	(21)	43,789	(93.3)	3136	(6.7)	36,454	(77.7)	10,471	(22.3)
CTA	86,184	(38)	68,688	(79.7)	17,496	(20.3)	56,993	(66.1)	29,191	(33.9)
MRA	76,551	(34)	56,274	(73.5)	20,277	(26.5)	47,351	(61.9)	29,200	(38.1)

Unless otherwise indicated, percentages of total values refer to the row. n. a. = not available. M, Q1/3 = median with first/third quartile. CEA = carotid thrombendarteriectomy. CAS = carotid artery stenting. CTA = computed tomography angiography. MRA = magnetic resonance angiography.

**Table 3:** Neurological assessment by treatment and indication group.

	Overall (Column-%)		Pre-Operative Neurological Assessment				Post-Operative Neurological Assessment			
			Yes		No		Yes		No	
**Asymptomatic (A)**										
CEA	100,915	(82)	58,163	(57.6)	42,752	(42.4)	53,286	(52.8)	47,629	(47.2)
CAS	21,448	(18)	15,262	(71.2)	6186	(28.2)	13,294	(62.0)	8154	(38.0)
**Symptomatic (elective, B)**										
CEA	65,852	(83)	58,356	(88.6)	7496	(11.4)	45,526	(69.1)	20,326	(30.9)
CAS	13,115	(17)	12,053	(91.9)	1062	(8.1)	11,125	(84.8)	1990	(15.2)
**Symptomatic (emergency, C1)**										
CEA	6076	(61)	5461	(89.9)	615	(10.1)	4631	(76.2)	1445	(23.8)
CAS	3886	(39)	3745	(96.4)	141	(3.6)	3658	(94.1)	228	(5.9)
**Simultaneous procedure (C2)**										
CEA	3802	(55)	1734	(45.6)	2068	(54.4)	1482	(39.0)	2320	(61.0)
CAS	3049	(45)	2656	(87.1)	393	(12.9)	2581	(84.7)	468	(15.3)
**Others (C3)**										
CEA	6881	(69)	5319	(77.3)	1562	(22.7)	4445	(64.6)	2436	(35.4)
CAS	3109	(31)	2710	(87.2)	399	(12.8)	2591	(83.3)	518	(16.7)

Unless otherwise indicated, percentages of total values refer to the row. CEA = Carotid thrombendarteriectomy. CAS = carotid artery stenting. Group A (asymptomatic patients), Group B (symptomatic patients receiving elective treatment), Group C (symptomatic patients receiving emergency treatment (C1), simultaneous operations (C2), other procedures (C3)).

**Table 4:** Primary outcome rate by treatment and neurological assessment. Percentages in parentheses indicate risk of any stroke or death until discharge (POE).

	Overall		Pre-Operative Neurological Assessment					Post-Operative Neurological Assessment				
			Yes		No		*p*	Yes		No		*p*
**Any stroke or death**												
**Overall**	6254	(2.74)	4978	(3.01)	1276	(2.04)	**<0.001**	5331	(3.74)	923	(1.08)	**<0.001**
**Asymptomatic (A)**												
CEA	1461	(1.45)	883	(1.52)	578	(1.35)	**0.031**	1169	(2.19)	292	(0.61)	**<0.001**
CAS	374	(1.74)	278	(1.82)	96	(1.55)	0.191	315	(2.37)	59	(0.72)	**<0.001**
**Symptomatic (elective, B)**												
CEA	1838	(2.79)	1660	(2.84)	178	(2.37)	**0.022**	1608	(3.53)	230	(1.13)	**<0.001**
CAS	485	(3.70)	438	(3.63)	47	(4.43)	0.220	458	(4.12)	27	(1.36)	**<0.001**
**Symptomatic (emergency, C1)**												
CEA	414	(6.81)	357	(6.54)	57	(9.27)	**0.014**	364	(7.86)	50	(3.46)	**<0.001**
CAS	417	(10.7)	401	(10.7)	16	(11.3)	0.918	393	(10.7)	24	(10.5)	1.000
**Simultaneous procedure (C2)**												
CEA	313	(8.23)	139	(8.02)	174	(8.41)	0.700	188	(12.7)	125	(5.39)	**<0.001**
CAS	318	(10.4)	287	(10.8)	31	(7.89)	0.093	281	(10.9)	37	(7.91)	0.063
**Others (C3)**												
CEA	386	(5.61)	311	(5.85)	75	(4.80)	0.130	328	(7.38)	58	(2.38)	**<0.001**
CAS	248	(7.98)	224	(8.27)	24	(6.02)	0.147	227	(8.76)	21	(4.05)	**<0.001**

CEA = Carotid thrombendarteriectomy. CAS = carotid artery stenting. Group A (asymptomatic patients), Group B (symptomatic patients receiving elective treatment), Group C (symptomatic patients receiving emergency treatment (C1), simultaneous operations (C2), other procedures (C3)).

## Data Availability

The datasets analyzed during the current study are available on request from the IQTIG, https://iqtig.org/qs-verfahren-uebersicht/sekundaere-datennutzung/.

## Supplementary Materials

The following supporting information can be downloaded at: https://www.mdpi.com/article/10.3390/jcm13144177/s1, Table S1: Hospital and regional characteristics for patients by neurological assessment (Bavarian data). Table S2: Outcome rates of patients by neurological assessment. Figure S1: Multivariable regression analysis of determinants associated with preprocedural neurologic examination. Figure S2: Multivariable regression analysis of determinants associated with postprocedural neurologic examination.
