# Supplementary material for: Determinants of Pre- and Post-Procedural Neurological Assessment, and Outcome of Carotid Endarterectomy or Stenting
Source: J Clin Med. 2024 Jul 17;13(14):4177. doi: 10.3390/jcm13144177 (PMC11278376; doi:10.3390/jcm13144177)

# - Supplementary Data -

Determinants of pre- and post-procedural neurological assessment, and outcome of carotid endarterectomy or stenting.

– Secondary data analysis of the German statutory quality assurance database –

Michael Kallmayer, MD, Christoph Knappich, MD, Felix Kirchhoff, MD,
Bianca Bohmann, M.Sc., Vanessa Lohe, B.Sc., Shamsun Naher, M. Sc.
Hans-Henning Eckstein MD, PhD, Andreas Kuehnl, MD, MPH, MBA,

1. Department for Vascular and Endovascular Surgery, Klinikum rechts der Isar, Technical University of Munich, Munich, Germany

Total number of tables: 2

Total number of figures: 2

Supplement:

Supplemental Table 1: Hospital and regional characteristics for patients by neurological assessment (Bavarian data).

	Overall	Pre-operative neurological assessment		Post-operative neurological assessment	
		Yes	No	Yes	No
**Type of hospital**					
Maximum care hospital	11780 (37)	8541 (72.5)	3239 (27.5)	7671 (65.1)	4109 (34.9)
Main care hospital	9070 (29)	6630 (73.1)	2440 (26.9)	5891 (65.0)	3179 (35.0)
Primary care hospital	3293 (10)	1662 (50.5)	1631 (49.5)	1153 (35.0)	2140 (65.0)
Specialized hospital	196 (1)	137 (69.9)	59 (30.1)	134 (68.4)	62 (31.6)
Others	7454 (23)	3707 (49.7)	3747 (50.3)	2856 (38.3)	4598 (61.7)
**Type of hospital owner**					
Municipal / public	24641 (78)	17079 (69.3)	7562 (30.7)	13519 (54.9)	11122 (45.1)
Non-profit	1883 (6)	1140 (60.5)	743 (39.5)	1555 (82.6)	328 (17.4)
Private / for-profit	5269 (17)	2458 (46.7)	2811 (53.3)	2631 (49.9)	2638 (50.1)
**Hospital beds***					
Total	n. a.	545 (360–968)	543 (389–896)	545 360–905	706 (389–909)
Surgery	n. a.	145 (97–181)	130 (72–179)	51 40–73	56 (34–78)
Vascular surgery	n. a.	32 (25–41)	33 (25–72)	35 25–41	33 (25–72)
Neurology	n. a.	54 (40–75)	49 (34–78)	51 40–73	56 (34–78)
**On-site specialized units**					
DSG stroke unit	16226 (32)	11833 (72.9)	4393 (27.1)	11069 (68.2)	5157 (31.8)
DGG certified vascular center	8594 (17)	5762 (67.0)	2832 (33.0)	5313 (61.8)	3281 (38.2)
Emergency room	26272 (51)	17544 (66.8)	8728 (33.2)	14933 (56.8)	11339 (43.2)
**Settlement structure**					
Large city	14961 (47)	9133 (61.0)	5828 (39.0)	7594 (50.8)	7367 (49.2)
Urban county	2421 (8)	1772 (73.2)	649 (26.8)	1786 (73.8)	635 (26.2)
Rural county	5969 (19)	4746 (79.5)	1223 (20.5)	4316 (72.3)	1653 (27.7)
Sparsely populated county	8442 (27)	5026 (59.5)	3416 (40.5)	4009 (47.5)	4433 (52.5)
**GISD***	n. a.	0.34 (0.17–0.41)	0.35 (0.27–0.40)	0.32 (0.17–0.40)	0.36 (0.30–0.41)

If not stated otherwise, percentages refer to the row (row-%). * = median with first/third quartile. DSG = German stroke society; DGG = German society for vascular surgery and vascular medicine. GISD = standardized German Index of Socioeconomic Deprivation (measure to assess the degree of socioeconomic deprivation).

Supplemental Table 2: Outcome rates of patients by neurological assessment.

	Overall	Pre-operative neurological assessment			Post-operative neurological assessment		
		Yes	No	*p*	Yes	No	*p*
**Major stroke or death**							
**Asymptomatic**							
CEA	976 (0.97)	600 (1.03)	376 (0.88)	**0.016**	735 (1.38)	241 (0.51)	**<0.001**
CAS	213 (0.99)	162 (1.06)	51 (0.82)	0.131	173 (1.30)	40 (0.49)	**<0.001**
**Symptomatic (elective)**							
CEA	1403 (2.13)	1278 (2.19)	125 (1.67)	**0.004**	1201 (2.64)	202 (0.99)	**<0.001**
CAS	336 (2.56)	305 (2.53)	31 (2.92)	0.505	312 (2.80)	24 (1.21)	<0.001
**Symptomatic (emergency)**							
CEA	369 (6.07)	318 (5.82)	51 (8.29)	**0.019**	325 (7.02)	44 (3.04)	**<0.001**
CAS	388 (9.98)	373 (9.96)	15 (10.6)	0.904	365 (9.98)	23 (10.1)	1.000
**Simultaneous procedure**							
CEA	264 (6.94)	111 (6.40)	153 (7.40)	0.254	145 (9.78)	119 (5.13)	<0.001
CAS	294 (9.64)	268 (10.1)	26 (6.62)	**0.037**	258 (10.0)	36 (7.69)	0.142
**Others**							
CEA	314 (4.56)	252 (4.74)	62 (3.97)	0.226	262 (5.89)	52 (2.13)	<0.001
CAS	206 (6.63)	190 (7.01)	16 (4.01)	**0.032**	189 (7.29)	17 (3.28)	**0.001**
**Any postprocedural stroke**							
**Asymptomatic**							
CEA	1118 (1.11)	676 (1.16)	442 (1.03)	0.058	996 (1.87)	122 (0.26)	**<0.001**
CAS	302 (1.41)	226 (1.48)	76 (1.23)	0.175	271 (2.04)	31 (0.38)	**<0.001**
**Symptomatic (elective)**							
CEA	1369 (2.08)	1227 (2.10)	142 (1.89)	0.252	1259 (2.77)	110 (0.54)	**<0.001**
CAS	362 (2.76)	323 (2.68)	39 (3.67)	0.073	347 (3.12)	15 (0.75)	**<0.001**
**Symptomatic (emergency)**							
CEA	248 (4.08)	216 (3.96)	32 (5.20)	0.169	228 (4.92)	20 (1.38)	**<0.001**
CAS	188 (4.84)	184 (4.91)	4 (2.80)	0.353	180 (4.92)	8 (3.51)	0.421
**Simultaneous procedure**							
CEA	149 (3.92)	75 (4.33)	74 (3.58)	0.272	132 (8.91)	17 (0.73)	**<0.001**
CAS	151 (4.95)	140 (5.27)	11 (2.80)	**0.047**	138 (5.35)	13 (2.78)	**0.025**
**Others**							
CEA	289 (4.20)	233 (4.38)	56 (3.59)	0.192	262 (5.89)	27 (1.11)	**<0.001**
CAS	135 (4.34)	119 (4.39)	16 (4.01)	0.828	126 (4.86)	9 (1.74)	**0.002**

CEA = Carotid thrombendarteriectomy. CAS = carotid artery stenting. Group A (asymptomatic patients), Group B (symptomatic patients receiving elective treatment), Group C (symptomatic patients receiving emergency treatment (C1), simultaneous operations (C2), other procedures (C3).

Supplemental figure 1: Multivariable regression analysis of determinants associated with preprocedural neurologic examination

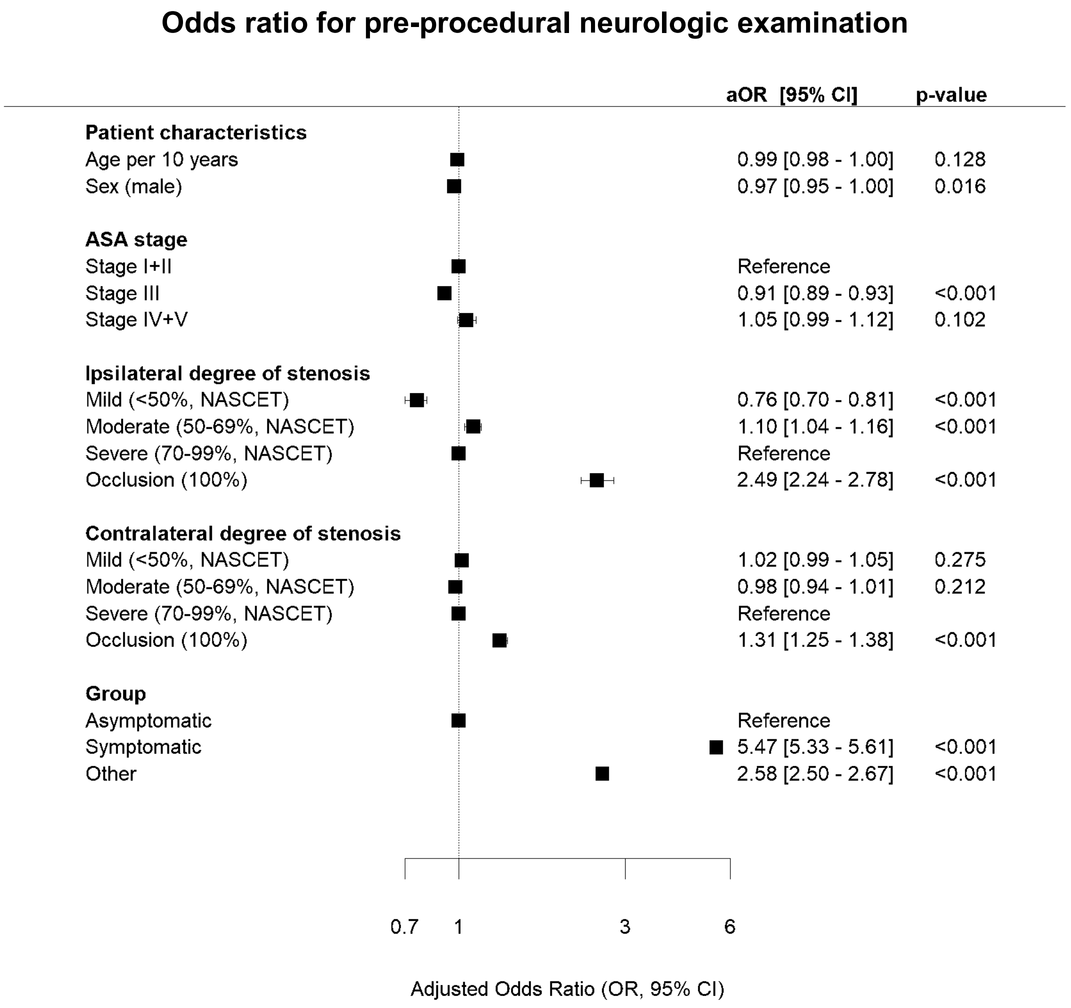

Supplemental figure 2: Multivariable regression analysis of determinants associated with postprocedural neurologic examination

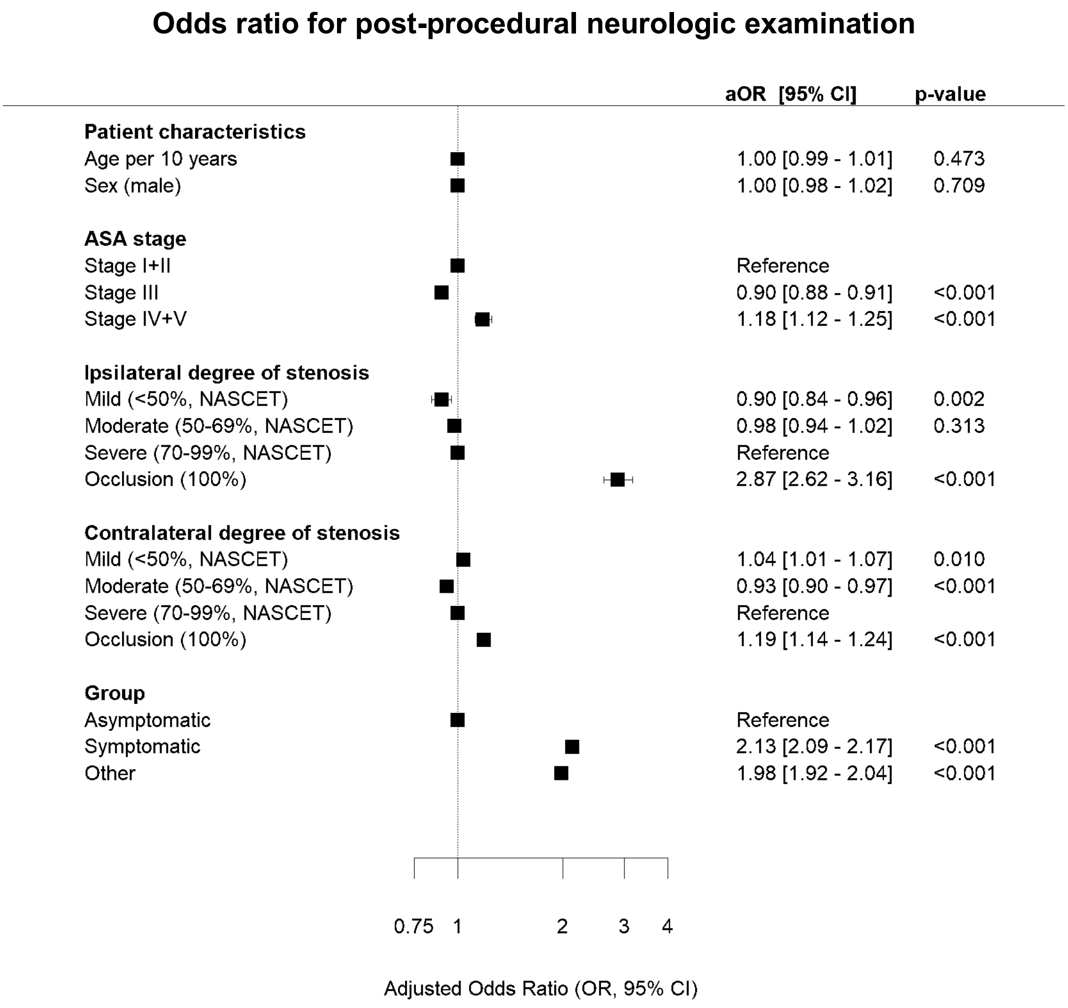

Supplement: Supplementary file 1 [file jcm-13-04177-s001.zip › jcm-3084642-supplementary.pdf]
